# Complete Coding Sequences of Three Members of the Kokobera Group of Flaviviruses

**DOI:** 10.1128/genomeA.00890-14

**Published:** 2014-09-18

**Authors:** David Warrilow, Sonja Hall-Mendelin, Jody Hobson-Peters, Natalie A. Prow, Richard Allcock, Roy A. Hall

**Affiliations:** a Public Health Virology Laboratory, Queensland Health Forensic and Scientific Services, Archerfield, Queensland, Australia; b Australian Infectious Diseases Research Centre, School of Chemistry and Molecular Biosciences, the University of Queensland, Queensland, Australia; c LotteryWest State Biomedical Facility–Genomics, School of Pathology and Laboratory Medicine, University of Western Australia, Perth, Western Australia, Australia; d Department of Clinical Immunology, Pathwest Laboratory Medicine WA, Royal Perth Hospital, Perth, Western Australia, Australia

## Abstract

The Kokobera group of flaviviruses circulates in Australia and Papua, New Guinea, and has been associated with occasional human polyarticular disease. To facilitate future studies to identify virulence determinants, the complete coding regions of the Stratford virus, and isolates of the Bainyik virus and Torres virus were obtained.

## GENOME ANNOUNCEMENT

The Kokobera group of flaviviruses (family, *Flaviviridae*; genus, Flavivirus) currently includes 5 candidate species: Kokobera (KOKV), Stratford (STRV), Bainyik (previously strain MK7979), Torres (previously strain TS5273), and New Mapoon (NMV) viruses (1). The prototype strain for this group, KOKV, was first isolated in 1960 in Mitchell River Mission in northern Queensland, Australia, from *Culex annulirostris* mosquitoes (2), and more recently from Sabai Island and Cape York Peninsula, Australia (3, 4). It has also been isolated in New South Wales and Western Australia (5) and the Northern Territory (6). STRV was isolated in 1961 from Cairns (2), the Bainyik virus in 1966 from Papua, New Guinea (PNG) (7), and the Torres virus in 2000 from Saibai Island in the Torres Strait, Queensland (8). NMV was isolated in northern Queensland in 1998 (8). Serum antibodies to these viruses have been detected in macropods and horses which may act as reservoir hosts (9–11).

KOKV and STRV have occasionally been associated with human polyarticular disease (9, 12–15). Experiments with laboratory mice indicated that the strain Bainyik virus has encephalitogenic potential (1). Genome sequencing would facilitate our understanding of virulence factors. Previous phylogenetic analyses based on partial genome sequence data indicated that the Bainyik virus formed a clade with the KOKV strain, the Torres virus formed a clade with the STRV strain, and NMV was more distantly related to these two clades (1). Currently, complete genome sequences exist only for KOKV and NMV.

Sequencing was as described in detail previously (16). Briefly, all strains were cultured in C6/36 mosquito cells. Culture fluid was centrifuged and filtered (0.2 µm) to remove contaminating material, treated with nucleases, and concentrated by ultra-centrifugation. Viral genomic RNA was extracted from the concentrated virus, and subjected to sequence-independent random amplification. The amplified material was then used to construct a library which was sequenced on a personal genome machine (PGM, Life Technologies). A consensus sequence was obtained using GeneiousPro 5.6 (17) by constructing contigs *de novo* followed by genome assembly using the KOKV genome sequence (GenBank accession no. NC_009029.2) as a scaffold. Any discrepancies in the sequence were resolved by construction of primer sets which spanned the region in question, followed by amplification and Sanger sequencing.

The nucleotide sequence length obtained varied (STRV, 10,560 nt; Bainyik virus, 10,648 nt; and Torres virus, 10,735 nt) mainly due to inefficiencies in the ability of the method to amplify the RNA genome termini. Nucleotide alignments with the complete 10874 nt KOKV genome sequence indicated that the Bainyik virus was more closely related to it than STRV or the Torres virus, as expected (nucleotide identity scores: KOKV-Bainyik virus, 80.4%; KOKV-STRV, 73.5%; KOKV-Torres virus, 74.3%). This was consistent with previous reports based on analyses of the untranslated regions (UTR) and envelope sequences (1). The putative complete open reading frame (ORF) was obtained for all three viruses (STRV, 3,414 aa; Bainyik virus, 3,410 aa; and Torres virus, 3,412 aa) which was similar to KOKV which has an ORF size of 3,410 aa.

### Nucleotide sequence accession numbers.

The GenBank accession numbers for the virus sequences are STRV (KM225263), Bainyik virus (KM225264), and Torres virus (KM225265).
